# Patent foramen ovale and stroke: Does presence of a migraine headache or any character of patent foramen ovale increase the risk of stroke?

**Published:** 2015-01-05

**Authors:** Abdolhamid Shariat, Ehsan Yaghoubi, Kamran Aghasadeghi, Abbas Rahimi, Reza Nemati, Nahid Ashjazadeh

**Affiliations:** 1 Department of Neurology, Shiraz Neurosciences Research Center AND Clinical Neurology Research Center, Shiraz University of Medical Sciences, Shiraz, Iran; 2 Department of Neurology, Shiraz Medical School, Shiraz University of Medical Sciences, Shiraz, Iran; 3 Department of Neurology, Shiraz Neurosciences Research Center, Shiraz University of Medical Sciences, Shiraz, Iran; 4 Department of Neurology, Clinical Neurology Research Center, Department of Neurology, Shiraz University of Medical Sciences, Shiraz, Iran; 5 Department of Cardiology, Shiraz Medical School, Shiraz University of Medical Sciences, Shiraz, Iran; 6 Department Neurology, Shiraz Neurosciences Research Center, Shiraz University of Medical Sciences, Shiraz, Iran

**Keywords:** Patent Foramen Ovale, Stroke, Migrain, Cerebrovascular

The etiology of ischemic stroke remains unidentified by routine diagnostic testing in about 40% of patients.^1^ Patent foramen ovale (PFO) has been proposed as a possible cause of paradoxical cardioembolism and is found in 27% of unselected adults.^2^ Nevertheless, the specific category of PFO bearers, who are prone to ischemic stroke, remains unidentified.^3^ This study aims to determine the differences of some characters of PFO between patients with ischemic stroke in whom PFO was associated with another major source of stroke and those with PFOs as the primary cause of ischemic stroke. We compared and contrasted transesophageal echocardiography (TEE) and transcranial Doppler sonography findings between the patients with cryptogenic stroke and the patients with stroke of determined cause. A sunray transcranial Doppler ultrasound device version FD-T98II (Guangzhou Doppler Electronic Technologies, China) was used in all the patients. The device was set to a small sample volume of 10 mm in length and minimum possible gain to provide a setting optimal for micro-embolic signal (MES) discrimination from the background spectrum. The MES was defined as typical visible and audible (chirp, click), short duration (0.1 s) and high-intensity signals within the Doppler flow spectrum. The number of MES was counted using Valsalva maneuver (VM) and was graded as 0, І, ІІ or ІІІ if 0, 1-9, 10-49 or ≥ 50 MES, respectively, were detected. TEE was performed to identify any potential cardiac source of embolism. Diagnosis of PFO was based on the presence of at least 3 bubbles in the left atrium after 4 cardiac cycles of the right atrium became opaque with contrast bubbles. The shunt was graded as І, ІІ, or ІІІ if 3-9, 10-49, or ≥ 50 bubbles, respectively, were visualized in the left atrium. The maximal diameter of PFO was measured in the same view. Those PFOs with a diameter < 2 mm were considered as small, 2-4 mm as moderate, and ≥ 4 mm as large. In addition, we compared the conventional risk factors for ischemic stroke and the presence of migraine headache (MH) between these two groups.

Seventy-eight consecutive patients with PFO on TEE examination who had stroke for the first time, who presented with clinical signs of rapidly developing focal cerebral dysfunction and were admitted to a stroke center in Namazi Hospital, Shiraz, Iran, were recruited for this study. Infarct etiology was classified according to the Trial of Org 10172 in Acute Stroke Treatment criteria.^4^ Then, the patients with two or more causes of stroke other than PFO to the stroke of determined cause group and those who had PFO with or without atrial septal aneurysm (ASA) without any other causes for stroke to the cryptogenic stroke group were assigned.

Thirty-one patients (39.7%) had cryptogenic strokes and 47 (60.3%) had strokes of determined causes. There was no statistically significant difference between these groups with respect to sex, conventional risk factors for ischemic stroke, history of VM prior to stroke. In addition, there was no statistically significant difference in the presence of ASA, amount of cardiac shunt, PFO size and amount of MES between the groups. Patients with cryptogenic stroke were significantly younger (57.4 ± 13.9 vs. 65.1 ± 13.7, P = 0.018) and had less prior history of ischemic heart disease (6.5 vs. 27.7%, P = 0.020). Using the ROC curve, the cut-off point for the patients with cryptogenic stroke was less than 57 years of age.

MH was more prevalent in the patients with cryptogenic stroke, but the difference was not statistically significant (29% vs. 19.6%, P = 0.330). In the subgroup of patients with cryptogenic stroke, hypertension and ASA were significantly less frequent (P = 0.001 and P = 0.013, respectively) and MH was significantly more frequent (P = 0.028; odds ratio, 3.3; 95% confidence interval, 1.1-9.8). MH was significantly more prevalent among females in this age group (P = 0.004; odds ratio, 5.4; 95% confidence interval, 1.6-18.8). None of the patients with MH reported previous ischemic heart disease (P = 0.017), and none of them who were younger than 57 years of age were taking anti-platelet medications prior to stroke onset. Our findings suggest that PFO may play a more significant role in the pathogenesis of ischemic stroke in patients younger than 57 years of age. Patients with MH did not have bigger PFOs (P = 0.780), more cardiac shunting (P = 0.980) or more MES (P = 0.420), when compared to patients without past histories of MH.

We did not compare the characters of PFO in patients with or without MH and in patients younger than 57 years of age. Subjects with PFO who were suffering from MH and are younger than 57 years of age, especially in females may benefit from stroke-prevention measures. However, this hypothesis needs to be confirmed in future case-controlled clinical trials.

## Notes

**How to cite this article: **Shariat A, Yaghoubi E, Aghasadeghi K, Rahimi A, Nemati R, Ashjazadeh N. Patent foramen ovale and stroke: Does presence of a migraine headache or any character of patent foramen ovale increase the risk of stroke? Iran J Neurol 2015; 14(1): 50-1.
